# A tumor-infiltrating B lymphocytes -related index based on machine-learning predicts prognosis and immunotherapy response in lung adenocarcinoma

**DOI:** 10.3389/fimmu.2025.1524120

**Published:** 2025-03-24

**Authors:** Jiale Fang, Siyuan Yu, Wei Wang, Cheng Liu, Xiaojia Lv, Jiaqi Jin, Xiaomin Han, Fang Zhou, Yukun Wang

**Affiliations:** ^1^ Department of Pharmacology, Southern University of Science and Technology, Shenzhen, China; ^2^ Department of Pharmacology, Air Force Medical University, Xi’an, China; ^3^ Department of Pharmacy, Southern University of Science and Technology Hospital, Shenzhen, China; ^4^ Department of Basic Medicine and Law, Baotou Medical College, Baotou, China

**Keywords:** lung cancer, immunotherapy, machine learning, TCGA, B-cell, tumor microenvironment

## Abstract

**Introduction:**

Tumor-infiltrating B lymphocytes (TILBs) play a pivotal role in shaping the immune microenvironment of tumors (TIME) and in the progression of lung adenocarcinoma (LUAD). However, there remains a scarcity of research that has thoroughly and systematically delineated the characteristics of TILBs in LUAD.

**Method:**

The research employed single-cell RNA sequencing from the GSE117570 dataset to identify markers linked to TILBs. A comprehensive machine learning approach, utilizing ten distinct algorithms, facilitated the creation of a TILB-related index (BRI) across the TCGA, GSE31210, and GSE72094 datasets. We used multiple algorithms to evaluate the relationships between BRI and TIME, as well as immune therapy-related biomarkers. Additionally, we assessed the role of BRI in predicting immune therapy response in two datasets, GSE91061 and GSE126044.

**Result:**

BRI functioned as an independent risk determinant in LUAD, demonstrating a robust and reliable capacity to predict overall survival rates. We observed significant differences in the scores of B cells, M2 macrophages, NK cells, and regulatory T cells between the high and low BRI score groups. Notably, BRI was found to inversely correlate with cytotoxic CD8+ T-cell infiltration (r = -0.43, p < 0.001) and positively correlate with regulatory T cells (r = 0.31, p = 0.008). We also found that patients with lower BRI were more likely to respond to immunotherapy and were associated with reduced IC50 values for standard chemotherapy and targeted therapy drugs, in contrast to higher BRI. Additionally, the BRI-based survival prediction nomogram demonstrated significant promise for clinical application in predicting the 1-, 3-, and 5-year overall survival rates among LUAD patients.

**Discussion:**

Our study developed a BRI model using ten different algorithms and 101 algorithm combinations. The BRI could be a valuable tool for risk stratification, prognosis, and selection of treatment approaches.

## 1 Introduction

In 2022, it was estimated that there were 19.97 million new cancer cases and 9.74 million cancer deaths globally. Among these, lung cancer had the highest incidence with 2.481 million new cases, accounting for 12.4% of all new cancer cases worldwide. This makes lung cancer the leading cancer globally once again, after being surpassed by breast cancer in 2020. Furthermore, lung cancer remains the most deadly cancer, constituting 18.7% of all cancer-related deaths (1). Lung tumors are classified into two broad categories by the World Health Organization (WHO): non-small cell lung cancer (NSCLC), which comprises 80-85% of all lung cancer cases, and small cell lung cancer (SCLC), which accounts for the remaining 15% of cases (2). Despite the increased chances of successful treatment and prognosis with early detection, lung cancer remains fatal due to challenges such as the lack of satisfactory prognostic markers, drug resistance, metastasis, and genetic heterogeneity (3). Lung cancer is characterized as an immunogenic cancer marked by chronic inflammation (4). The interactions between cancer cells and the tumor immune microenvironment play an irreplaceable role in tumor progression, metastasis, and treatment. While current immunotherapy research primarily focuses on T cells, growing evidence suggests that TILBs also play a crucial synergistic role in tumor control (5). TILBs are a fundamental component of the tumor immune microenvironment (6). Typically, TILBs do not function alone but are closely related to T cells and myeloid cells. In most immunologically “hot” tumors, TILBs are present at levels significantly higher than in healthy non-lymphoid tissues. In fact, exhausted or dysfunctional CD8 and CD4 TILs often express the B cell-recruiting chemokine C-X-C motif ligand 13 (CXCL13), indicating that they are programmed to seek assistance from TILBs in response to persistent tumors (7). This interaction ultimately leads to the formation of tertiary lymphoid structures (TLSs), newly formed lymph node-like structures within the tumor stroma that appear to actively participate in initiating and maintaining adaptive immune responses. Similar to T cells, TIL-TILBs are associated with positive prognostic value in most cancers, and they can significantly enhance the prognostic impact of CD4 and CD8 TILs, especially in tumors containing TLSs (8). In summary, TILBs play an indispensable role in the tumor immune microenvironment and hold great potential for immunotherapy. However, the role of TILBs in LUAD remains underexplored, and the mechanisms by which TILBs participate in immunotherapy have yet to be fully elucidated. Based on machine learning algorithms, pathological features can serve as potential prognostic biomarkers for renal cell carcinoma (9, 10). Our research endeavors to integrate single-cell sequencing, transcriptomic profiling, and other multi-omics approaches, augmented by the application of machine learning—a burgeoning and potent tool in prognostic modeling—to elucidate the characteristic genes of TILBs. Through this integrative approach, we aim to delineate the functional roles of TILBs in the immune microenvironment and assess their potential significance in the context of immunotherapy. Our results may provide further evidence for the critical role of TILBs in the prognosis and treatment of LUAD.

## 2 Materials and methods

### 2.1 Dataset acquisition and processing

**Figure 1:**
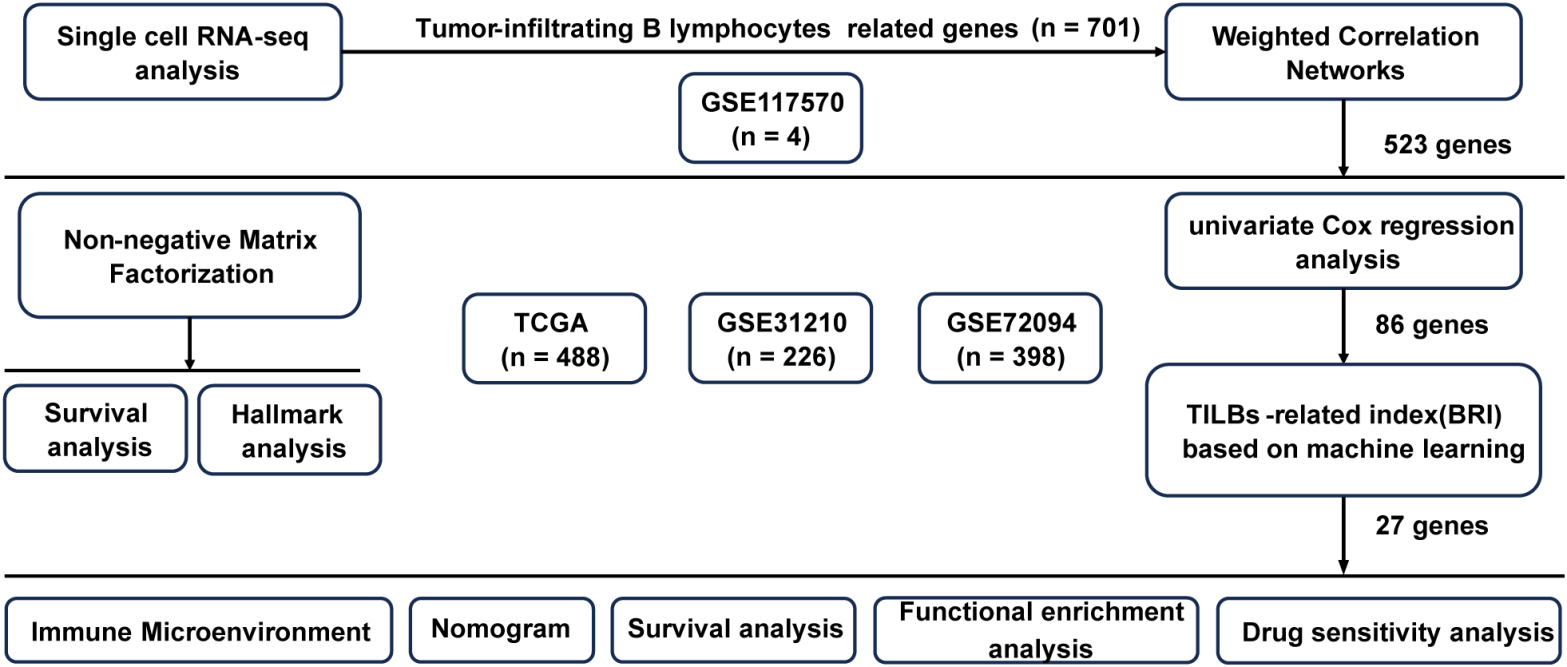
The flowchart of research.

**Figure 1** shows the flowchart of our study. Data on single-cell expression were sourced from the Gene Expression Omnibus (GEO) database, particularly from the GSE117570 dataset (n = 4). Data on RNA sequencing and LUAD genomic mutations were sourced from The Cancer Genome Atlas (TCGA, n = 488) database. Furthermore, the LUAD prognostic model was validated using three separate public datasets: GSE31210 (n =226) (11), and GSE72094 (n = 398) (12). Expression data from the TCGA and all GEO datasets underwent normalization via the “sva” package (13) prior to additional analysis. To explore the relationship between the BRI and response to immune checkpoint blockade (ICB) therapy, we analyzed two datasets with documented immunotherapy outcomes: GSE91061 (14) and GSE126044 (15). Data on drug sensitivity were sourced from the Genomics of Drug Sensitivity in Cancer (GDSC) website (https://www.cancerrxgene.org/).

### 2.2 Analysis of single-cell RNA-seq

The scRNA-seq data were processed using the “Seurat” R package (16), a dedicated R toolkit for single-cell genomics. Genes identified in cells with fewer than three and fewer than 50 genes were omitted and the percentage of mitochondria was limited to less than 25%. The “LogNormalize” feature in the “Seurat” package was utilized to normalize expression data. In the initial phase of our analysis, principal component analysis (PCA) was conducted on the Seurat object utilizing the “RunPCA” function. To ascertain the optimal number of principal components (PCs) for subsequent analyses, the “ElbowPlot” function was employed. Subsequently, for dimensionality reduction and visualization, the UMAP algorithm (17) was applied. The parameter `dim` was set to include the top 15 PCs (dim = 1:15). Cell types were annotated with reference data from human primary cell atlases using the SingleR software package (18) To pinpoint the marker genes in each cluster, the “Seurat” package’s “FindAllMarkers” feature was employed (logFC = 0.5, Minpct = 0.25) (19). Genes associated with B-cell clusters were identified as markers related to TILBs.

### 2.3 Analysis of Weighted Correlation Networks

WGCNA (Weighted Gene Co-expression Network Analysis) (20) serves as a dependable instrument for delineating gene correlation patterns among various samples. WGCNA extends its focus beyond just differentially expressed genes, amalgamating data from myriad highly variable genes, aiding in pinpointing relevant gene groups and elucidating correlation studies with phenotypes. Utilizing the “WGCNA” package, we developed a scale-free coexpression network for TLBs-related markers, setting the β value at 0.85 to preserve some level of independence. Module identification utilized a dynamic tree-cutting method. For pinpointing gene clusters linked to LUAD’s clinical results, the module exhibiting the strongest positive and negative links with both survival duration and status was chosen for additional research.

### 2.4 Analysis of clustering in Non-negative Matrix Factorization

NMF (Non-negative Matrix Factorization) (21) clustering is capable of breaking down the initial matrix into various non-negative matrices, with stable clustering being attained by continuous decomposition and aggregation, thus identifying possible characteristics. Utilizing the gene sets from WGCNA’s chosen modules, the NMF algorithm applied the “NMF” package to assess if markers related to TLBs could identify varied patterns in LUAD patients (22). In this analysis, genes were submitted for NMF clustering based on their expression patterns. It was suggested that the optimal cluster count occurs at the initial K value, marking the start of a decline in the cophenetic coefficient, or at the point where the RSS curve exhibits an inflection point (23).

### 2.5 BRI derived from integrative methods in machine learning

Subsequently, potential predictive indicators within WGCNA modular gene collections were pinpointed through univariate Cox regression analysis. Subsequently, a unified BRI model was created integrating 10 machine-learning techniques and 101 different algorithmic combinations. The comprehensive algorithms encompassed Random Survival Forest (RSF), Elastic Network (Enet), Lasso, Ridge, Stepwise Cox, CoxBoost, Partial Least Squares Regression for Cox (plsRcox), Supervised Principal Components (SuperPC), Generalized Boosted Regression Modeling (GBM), and Survival Support Vector Machine (survival-SVM). Regarding model invocation, we adopted a “1 + 1” approach, where the first machine learning algorithm selects the feature variables, and the second machine learning algorithm builds the model based on these feature variables. The optimal modeling method is selected by comparing the C-index of each combined model evaluated on the test set. The TCGA cohort was equally divided into the training group and the internal validation group according to the relative proportion of survival status, whereas the test groups comprised GSE31210, and GSE72094.The Harrell concordance index (C-index) was computed across all groups. An ideal model was characterized by having the greatest average C-index and in cases where multiple models shared an identical average C-index, the TCGA cohort’s C-index was favored. The BRI score for each LUAD patient was determined by analyzing gene expression in the BRI and their respective coefficients. The patients in question were divided into groups with high and low BRI.

### 2.6 Assessment of the efficiency of BRI

Subsequently, comprehensive survival graphs were created for groups with low and high BRI. For evaluating the forecasting precision of BRI in LUAD, time ROC and clinical ROC curves were charted using the “timeROC” packages (24). Survival differences used log-rank testing with Kaplan-Meier curves. Multivariate Cox models adjusted for TNM stage, gender, age and BRI, reported through hazard ratios with 95% confidence intervals.

### 2.7 Assessment of immune microenvironment landscape based on BRI

The ESTIMATE (25), algorithm calculates a composite score of immune and stromal cells to reflect the overall immunogenicity of the tumor microenvironment, The CIBERSORT algorithm provides a detailed profile of immune cell infiltration but relies on the accuracy of the reference gene expression signature matrix. The QUANTISEQ algorithm infers the abundance of immune cells by analyzing the expression levels of specific gene modules, yet it requires high-quality preprocessing of the data. While xCell provides broad cell type coverage and robustness in immune infiltration analysis, it is limited in quantifying absolute cell proportions and identifying the presence of specific cell types. To overcome the limitations of individual algorithms, we integrated the results from multiple algorithms to provide a more comprehensive assessment of the immune microenvironment Utilizing the “immunedeconv” package (26), three distinct algorithms (CIBERSORT, QUANTISEQ, XCELL) were employed to measure the varying percentages of immune cells infiltrating the system. The relationship between BRI/ts and immune cells was examined using Spearman’s rank correlation analysis. Furthermore, the quantity of immune cells and their activity or function scores were assessed through a single-sample gene set enrichment analysis (ssGSEA) using the “GSVA” package (27). Following this, the “ggpubr” package(https://cran.r-project.org/web/packages/ggpubr/) was utilized to contrast the expression rates of shared immune checkpoints and genes related to human leukocyte antigen (HLA) between groups with high and low BRI.

### 2.8 Assessment of therapeutic benefits BRI-based

We utilized two datasets with immunotherapy outcomes, GSE91061 and GSE126044, to investigate the relationship between the BRI and immune checkpoint blockade (ICB) treatment response. Using our machine learning model, we assigned BRI to patients in both datasets and stratified them into high-BRI and low-BRI groups. We then compared the proportions of stable disease (SD) and progressive disease (PD) versus complete response (CR) and partial response (PR) between the two groups. The “oncoPredict” package was utilized to compute the IC50 values, signifying drug sensitivity, where lower values denote increased sensitivity. Data on the three-dimensional configurations of chemotherapy and specific treatment targets were sourced from the PubChem database. Within the GSE91061 group, patients were classified according to their treatment reaction: those showing partial (PR) and complete (CR) response were deemed responders, whereas individuals with progressive (PD) and stable (SD) disease were labeled as non-responders. The “ggpubr” package was employed to assess the differences in IPS, TIDE score, and IC50 values among groups with high and low BRI.

### 2.9 Statistical analysis

Every statistical evaluation was conducted utilizing R software (version 4.4.0). To evaluate categorical variables, the χ2 test was utilized, while continuous variables underwent comparison through the Wilcoxon rank-sum test (applicable to samples with non-normal distribution and varying variances) or the T test (applicable to both sets of samples with normal distribution and identical variance). To assess the relationship between two continuous variables, Pearson’s rank correlation analysis was conducted. To evaluate the variance in Kaplan-Meier survival rates, the bi-directional log-rank test was employed.

## 3 Results

### 3.1 Single-cell analysis revealed cell subtypes an TILBs markers

**Figure 2:**
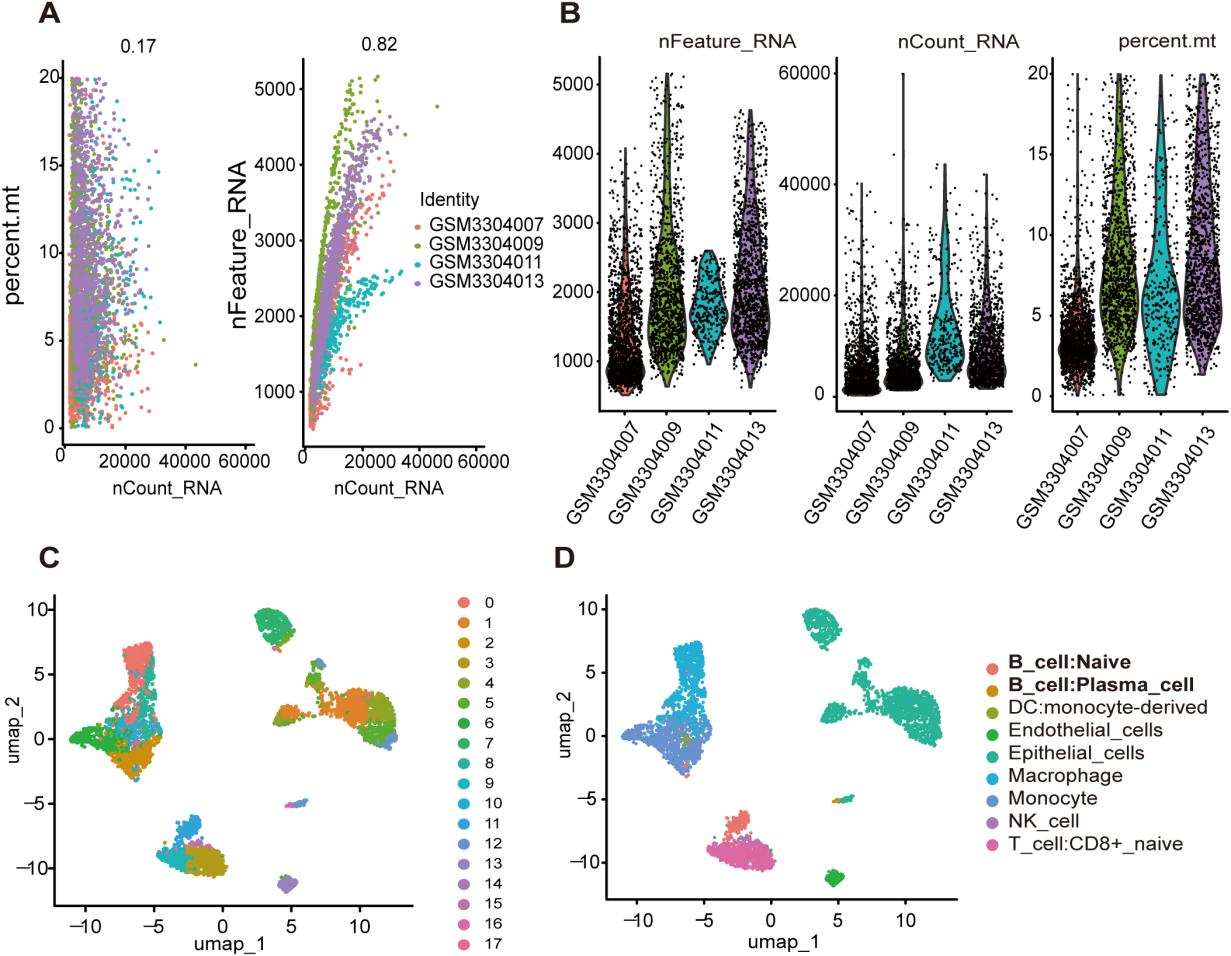
Identification of genes associated with B cells by single-cell data analysis. **(A)** Correlation analysis between nFeature_RNA\persent.mt and nCount_RNA after quality control. **(B)** The number of nFeature_RNA, nCount_RNA, and mitochondrial genes in each sample. **(C)** All cell samples were divided into 17 groups using UMP method. **(D)** Each group is annotated by SingleR method based on reference data from the Human Primary Cell Atlas.

Through rigorous quality control measures, we preprocessed the data and obtained 4,585 high-quality cell samples from four LUAD tissue samples. The number of sequenced genes exhibited a significant positive correlation with sequencing depth (**Figure 2A**). Following normalization, samples with either excessively low or high gene counts, as well as those with a disproportionately high percentage of mitochondrial genes, were excluded (**Figure 2B**). We selected the top 15 PCs before the inflection point, where the explained variance reaches a relatively stable plateau. These 15 PCs were chosen to capture the most significant sources of variation in the data while minimizing noise. For subsequent dimensionality reduction using UMAP, we set the parameter dim <- 1:15 to include these top 15 PCs. Additionally, we optimized the UMAP embedding parameters to a=0.9922 and b=1.112 to ensure a balanced trade-off between local and global structure preservation in the embedding. UMAP analysis identified 17 distinct clusters across all samples (**Figure 2C**). Subsequent cell subtype annotation was performed using SingleR with reference data from the Human Primary Cell Atlas (**Figure 2D**). We used the FeaturePlot function to display the distribution of characteristic markers for various cell types, which further validated the accuracy of cell annotation. (**Supplementary Figure S1**) The analysis revealed that clusters 11 and 16 were identified as TILBs. Utilizing the “FindAllMarkers” function of the Seurat package, we identified genes that were significantly highly expressed in the B cell clusters as TILBs markers. Additionally, a total of 701 marker genes were associated with TILBs (see **Supplementary Table 1**).

### 3.2 Pinpoint crucial modules linked to clinical results in LUAD

WGCNA utilized 701 TILBs genes, aiming to pinpoint essential survival modules, encompassing both the overall survival rate and duration in LUAD patients. An ideal power value of 11 for (β = 8) was ascertained. Following the establishment of the soft threshold, a coexpression network was developed, featuring (β = 0.85) (scale-free (R^2 = 0.920) (**Supplementary Figure S2A**). Five distinct modules were developed for subsequent examination. The connections among. A total of five modules were created for further analysis. The relationships between these modules were visualized (**Supplementary Figure S2B**), and a clustering dendrogram of the five modules is shown (**Supplementary Figure S2C**). The blue module, consisting of 104 genes, and the gray module, comprising 419 genes, exhibited the highest numbers of positive and negative correlations, respectively (see **Supplementary Table 2**).

### 3.3 NMF clustering identified four TILBs subtypes in LUAD

**Figure 3:**
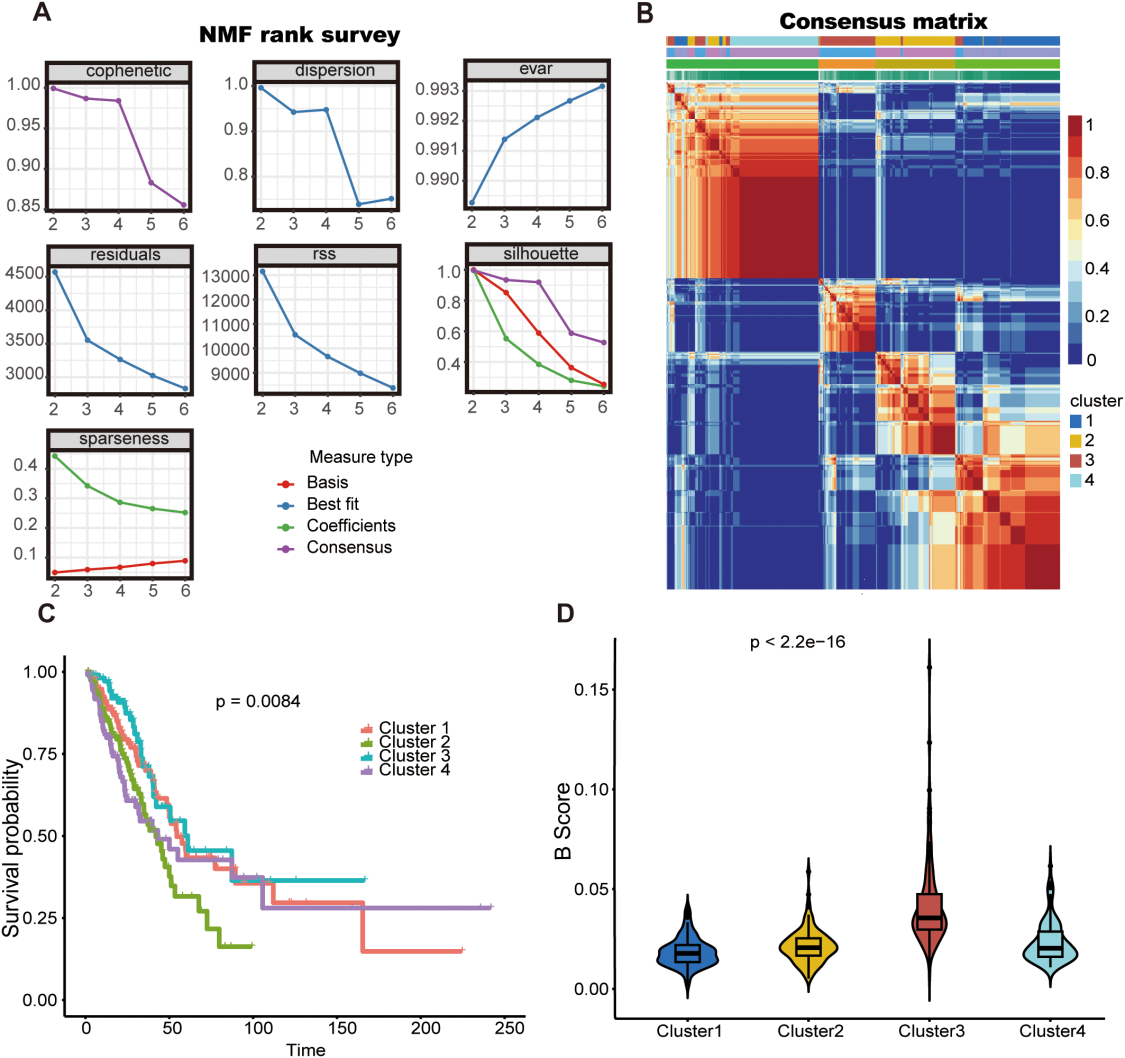
The NMF algorithm identified two TLBs-related subtypes in LUAD. **(A)** For the TCGA cohort, the cophenetic, dispersion, and sparseness distributions were evaluated across values of K = 2 to 6, K = 2was determined as the first value where the cophenetic coefficient showed a significant drop. **(B)** The consensus map shows gene expression across four clusters **(C)** Kaplan–Meier survival curves for the four identified clusters are shown for the TCGA cohort. **(D)** The violin diagram illustrates the difference in B-cell scores between the four clusters using the estimate algorithm.

Utilizing the expression profiles of 523 TILBs markers chosen by WGCNA, a clustering algorithm of NMF was employed to categorize the molecular variants of LUAD patients. The suggestion was made that the ideal cluster count ought to be the initial K value at which the cophenetic coefficient starts to significantly diminish. Ultimately, in the TCGA-LUAD dataset (**Figure 3A**), the ideal cluster number K was determined to be 4. The heatmap depicting the related consensus matrices within the TCGA dataset is depicted in **Figure 3B**. The overall survival rate for patients suffering from LUAD in cluster 3 was superior to those in clusters 1, 2, and 4 of the TCGA group (**Figure 3C**). Furthermore, the analysis of B cell scores in samples from four distinct clusters showed that patients in cluster 3 exhibited elevated B cell scores (**Figure 3D**). This additionally indicates the beneficial predictive function of TILBs in patients with LUAD.

### 3.4 Comprehensive development of a consensus BRI

**Figure 4:**
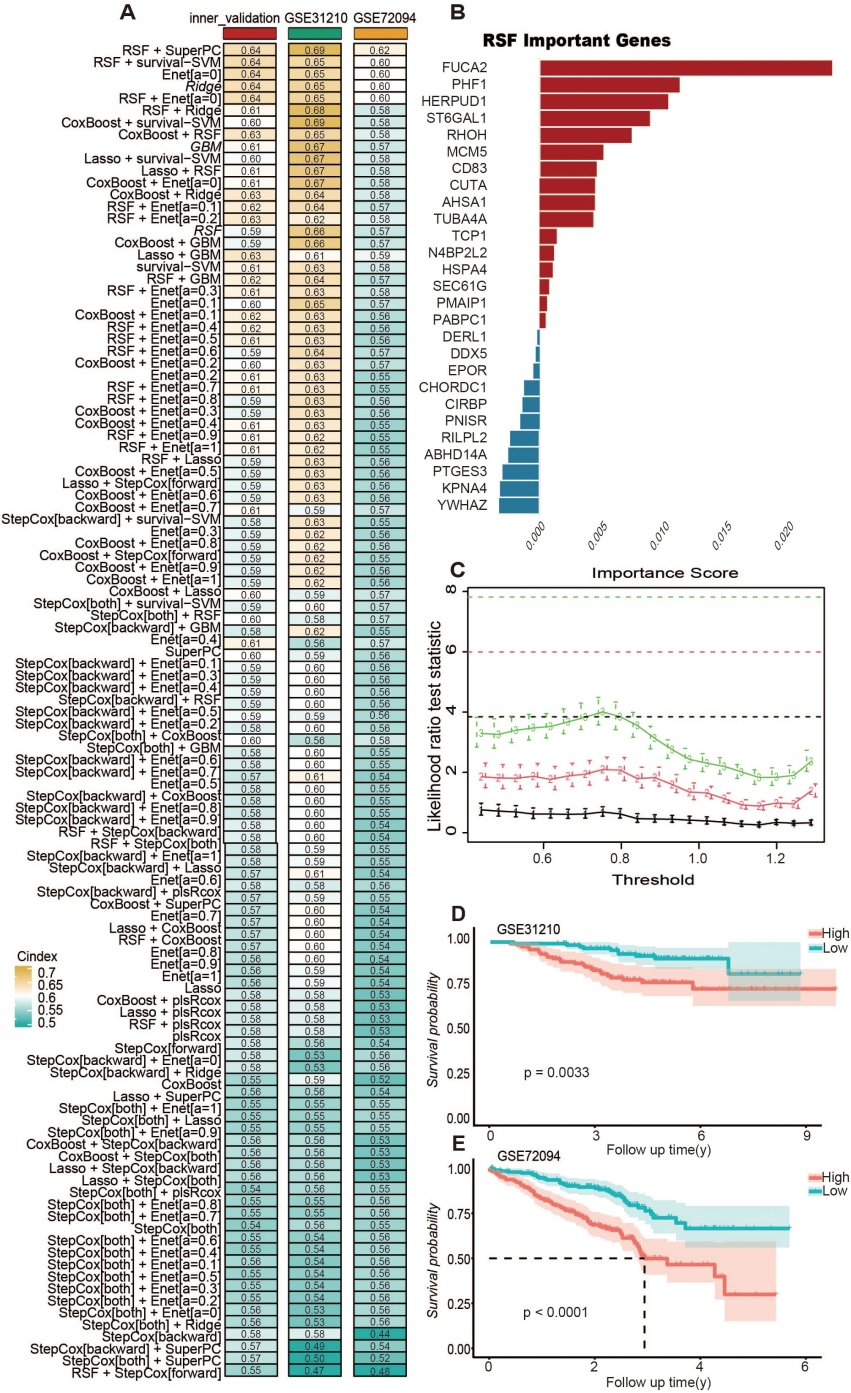
Integrative machine learning identified a consensus TLBs-related index (BRI). **(A)** The C-index of 101 kinds of prediction model based on combination of 10 machine learning algorithms. The combination of RSF and superPC produced the optimal model, achieving the highest average C-index of 0.67. **(B)** Through the RSF algorithm, 27 genes were screened as important variables for superPC modeling. **(C)** Use n.fold to determine the folds of cross-validation during the model building process in superPC. According to the final model, each sample obtained its own BRI score, which was divided into two groups: BRI high and BRI low according to the score. Kaplan–Meier survival curves for the two groups are shown in the GSE31210 cohort **(D)**, GSE72094 cohort **(E)**.

A univariate Cox analysis identified 86 genes possibly associated with TILBs, demonstrating a notable link with the prognosis of LUAD (see **Supplementary Table 3**). The TCGA-LUAD group segmented its data into training and internal validation categories based on inventory levels, to house 101 unique predictive models. For RSF, we utilized the default parameter settings (e.g., the number of trees was set to 1000, and the minimum number of samples required at each leaf node was set to 5). Feature importance scores were used to identify key genes. For LASSO regression, the optimal regularization parameter (λ) was determined through cross-validation to achieve feature selection and model fitting. For Coxboost, the selection coefficient of the CoxBoost model, where variables with a selection coefficient greater than 0 are considered. For other algorithms, parameter tuning was conducted based on 10-fold cross-validation to ensure model robustness and generalizability. In every model, the C-index was calculated for each group, with the results depicted in **Figure 4A**. Findings showed that the predictive model, created through the integration of RSF and superPC, was attaining the top average C-index of 0.65.Using RSF, we selected 27 key variables, including FUCA2, PHF1, TUBA4A, CUTA, N4BP2L2, ST6GAL1, YWHAZ, DERL1, AHSA1, CD83, PABPC1, SEC61G, MCM5, KPNA4, RHOH, DDX5, HSPA4, RILPL2, TCP1, ABHD14A, PMAIP1, HERPUD1, EPOR, PNISR, CHORDC1, PTGES3, and CIRBP **Figure 4B**). Further, we performed superPC modeling using these 27 genes and determined the optimal number of folds for cross-validation (**Figure 4C**). As expected, high BRI in LUAD patients were associated with poorer overall survival rates in the GSE31210 (**Figure 4D**) (p < 0.01) and GSE72094 (**Figure 4E**) (p < 0.0001) datasets.

### 3.5 Evaluation of the performance of BRI

**Figure 5:**
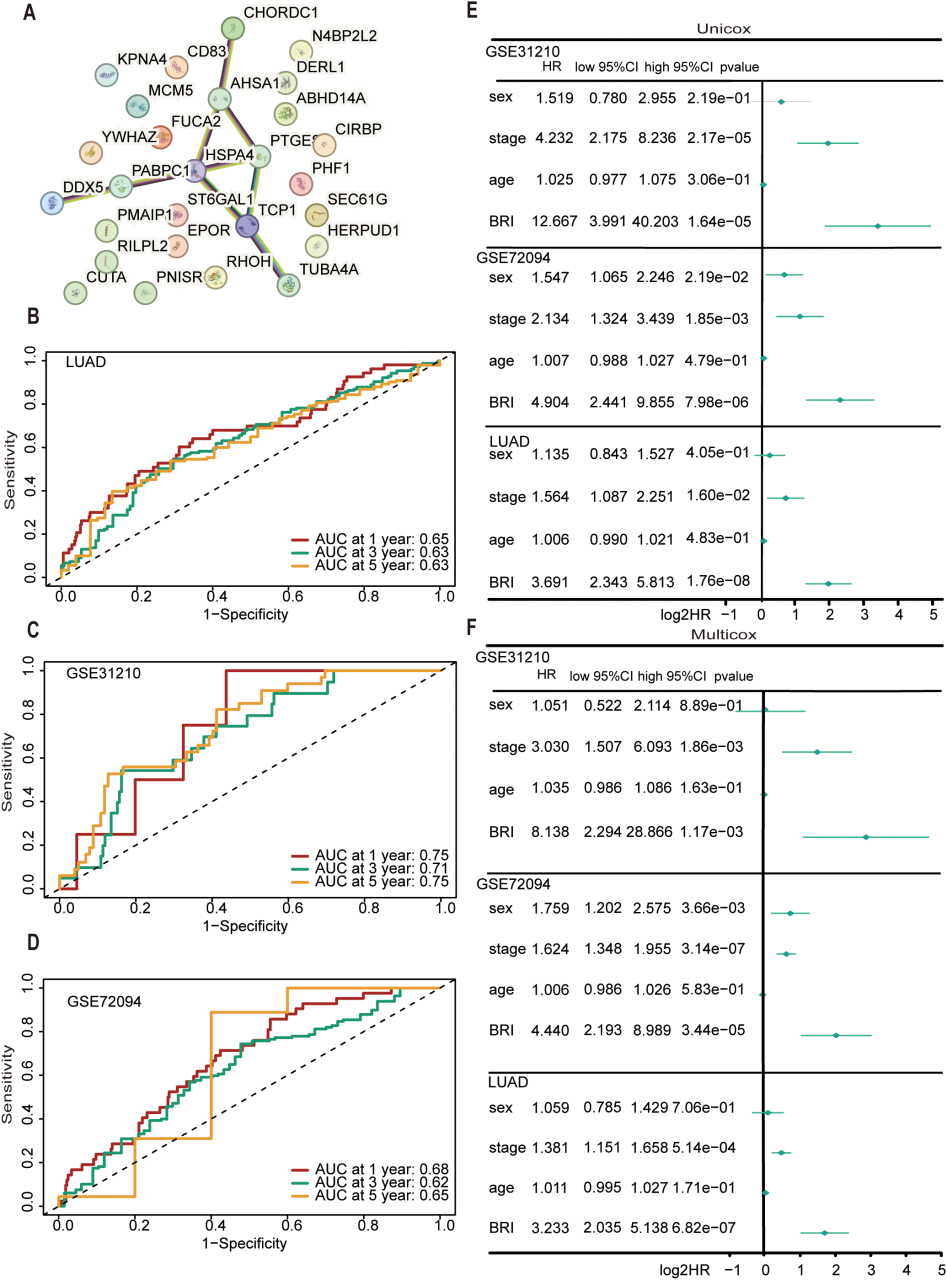
Evaluation of the performance of BRI. **(A)** The network node diagram illustrates the interaction between the 27 genes involved in model construction. TimeROC analysis evaluated the discrimination of BRI in the overall survival rate of patients with LUAD cohort **(B)**, GSE31210 cohort **(C)**, GSE72094 cohort **(D)**, with 1-, 3-, and 5-year. **(E)** Univariate ox regression analyses considering clinical parameters and BRI in the TCGA, GSE31210, and GSE72094 cohorts. **(F)** Multivariate Cox regression analyses considering clinical parameters and BRI in the TCGA, GSE31210, and GSE72094 cohorts.

We analyzed the interaction relationships among the 27 feature genes in the model (**Figure 5A**). Time ROC analysis assessed the discriminatory ability of BRI for overall survival in LUAD patients (**Figure 5B**), with 1-, 3-, and 5-year AUCs of 0.65, 0.63, and 0.63, respectively. In the GSE31210 cohort, the AUCs were 0.75, 0.71, and 0.75 (**Figure 5C**), while in the GSE72094 cohort, they were 0.68, 0.62, and 0.65 (**Figure 5D**). Moreover, both univariate and multivariate Cox regression analyses indicated that BRI is an independent risk factor for overall survival in the TCGA, GSE31210, and GSE72094 cohorts (**Figures 5E, F**).

### 3.6 Analyzing the tumor microenvironment through the lens of BRI

**Figure 6:**
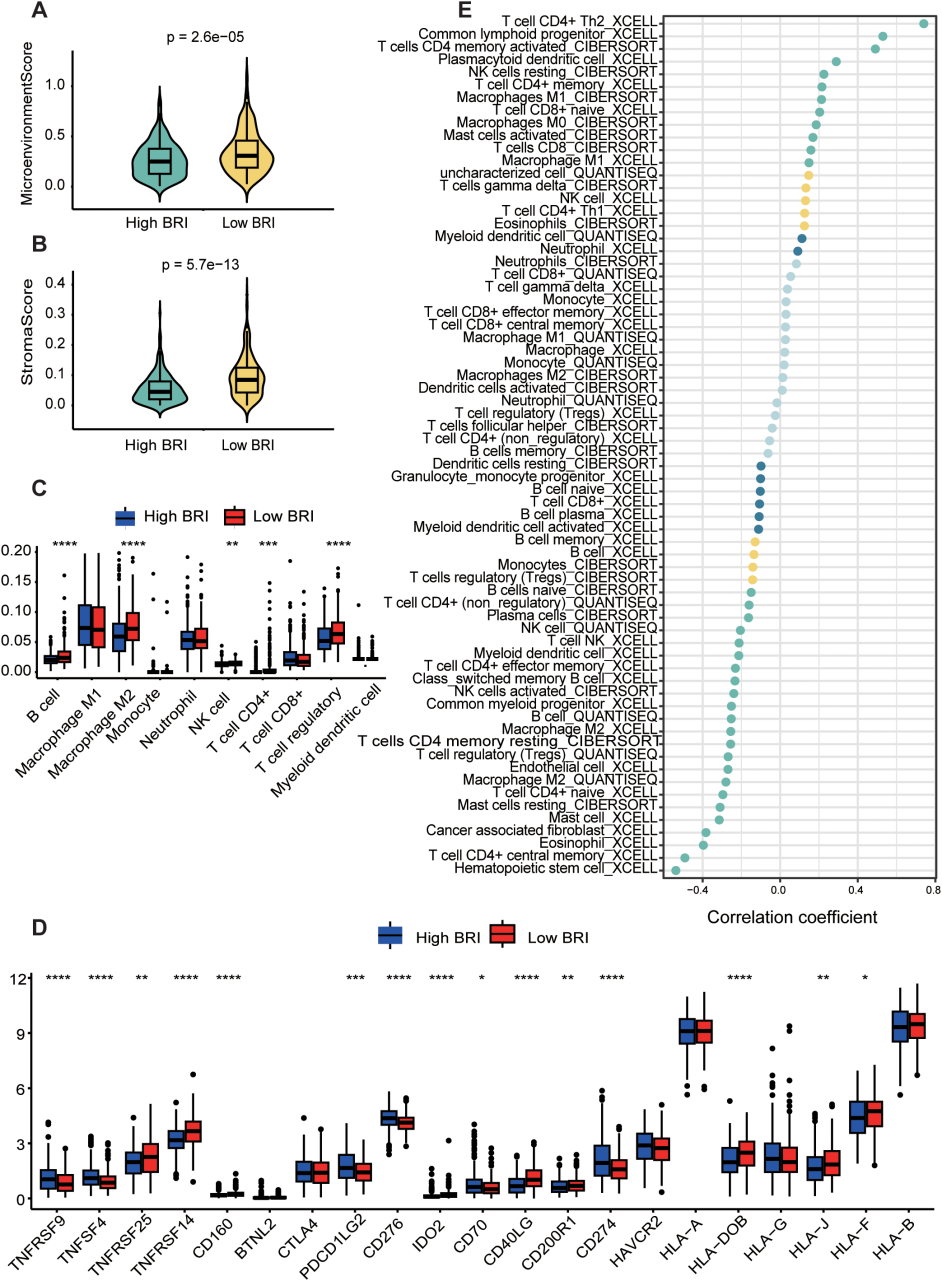
Dissection of the tumor microenvironment based on the BRI. LAUD patients with low BRI had higher Microenvionmentscore **(A)** and Stromascore **(B)** The score of each LUAD case were calculated with the Xcell algorithm. **(C)** The immune cell score of patients with LUAD was calculated using the estimate algorithm, and there was a difference between the two groups with high and low BRI. **(D)** The expression differences in immune checkpoints between the two groups with high and low BRI. **(E)** The correlation between BRI score and the abundance of immune cells was calculated using three algorithms, including CIBERSORT, QUANTISEQ, XCELL. *p < 0.05, **p < 0.01, ***p < 0.001, ****p<0.0001.

Using the xCell algorithm to estimate the microenvironment and stroma scores in LUAD patients, we found significant differences in microenvironment and stroma scores between patients with high and low BSI (**Figures 6A, B**). Subsequently, using three distinct algorithms (CIBERSORT, QUANTISEQ, XCELL) for immune cell scoring in LUAD patients, we also observed significant differences in the scores of B cells, M2 macrophages, NK cells, and regulatory T cells between the high and low BSI score groups (**Figure 6C**). Notably, BRI was found to inversely correlate with cytotoxic CD8+ T-cell infiltration (r = -0.43, p < 0.001) and positively correlate with regulatory T cells (r = 0.31, p = 0.008), highlighting the complex interplay within the immune microenvironment. To further investigate the correlation between BSI and tumor immunity in lung cancer, we analyzed the differences in the expression of immune markers between patients with high and low BRI. Significant differences were found in the expression levels of TNFRSF9, TNFSF4, TNFRSF25, TNFRSF14, CD160, PDCD1LG2, CD276, IDO2, CD70, CD40LG, CD200R1, CD274, HLA-DOB, HLA-J, and HLA-F between the two groups (**Figure 6D**). Lastly, we explored the correlation between BRI and the abundance of immune cells using several immune cell-related algorithms, which revealed that BRI were significantly negatively correlated with the abundance of most immune cells (**Figure 6E**) (see **Supplementary Table 4**). Additionally, through Gene Set Enrichment Analysis (GSEA), we identified that the differentially expressed genes between the two groups were primarily enriched in pathways related to B cell receptor signaling and Th1 and Th2 cell differentiation, among other tumor immune processes (**Supplementary Figure S3**).

### 3.7 BRI-based treatment strategy for LUAD

**Figure 7:**
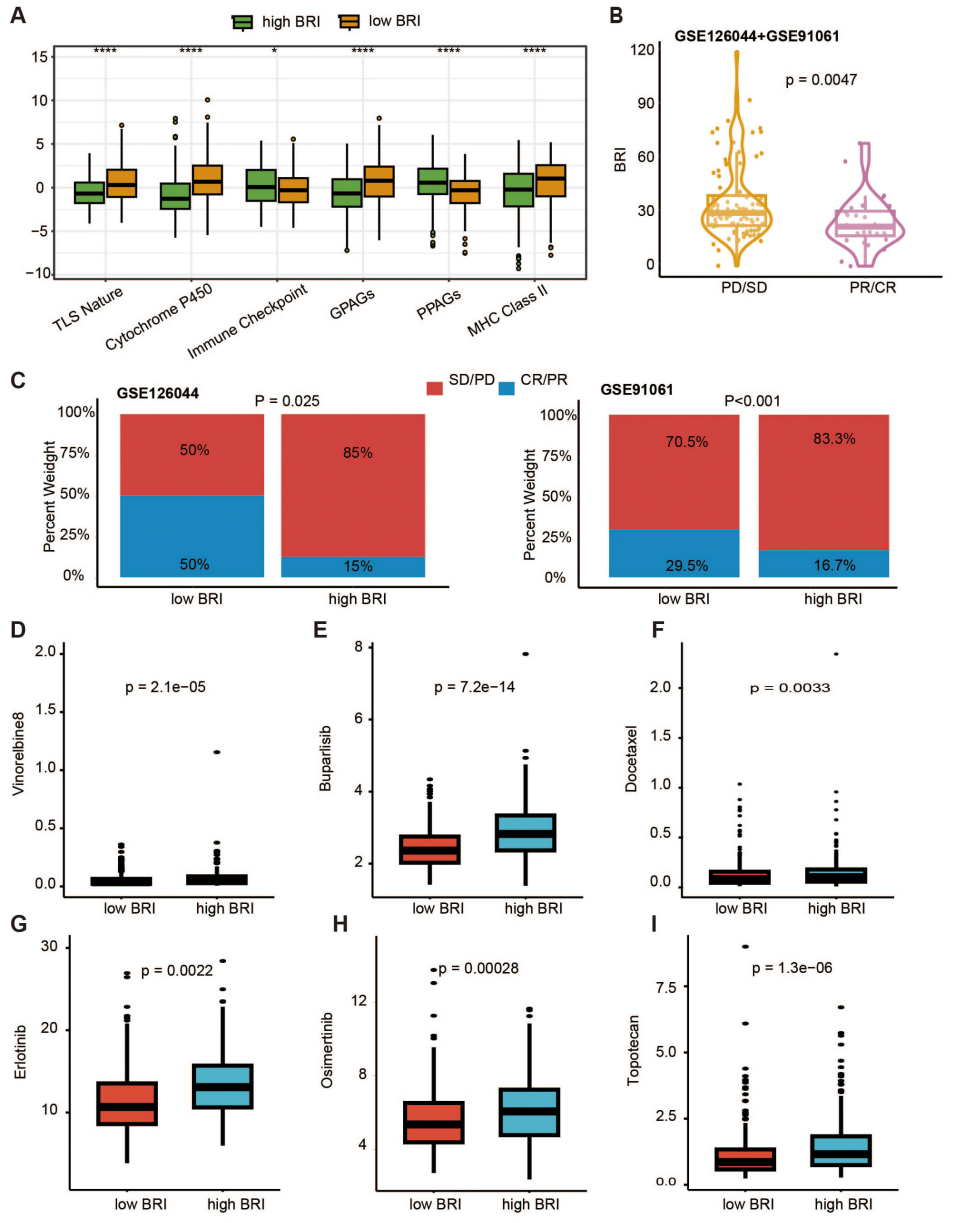
TLBs-related index (BRI)-based treatment strategy for LUAD. **(A)** Comparison of pharmacological indicators between the low BRI group and the high BRI group. **(B)** In the datasets GSE91061 and GSE126044, there was a significant difference in BRI between patients in the PD/SD group and those in the PR/CR group (p = 0.0047). **(C)** In the datasets GSE91061 and GSE126044, there were significant differences in the proportions of PD/SD and PR/CR between patients in the low BRI group and those in the high BRI group (GSE126044, p = 0.025; GSE91061, p < 0.001). The IC50 values of Buparlisib **(D)**, Docetaxel **(E)**, Erlotinib **(F)**, Osimertinib **(G)**, Topotecan **(H)**, Vinorelbine **(I)** were lower in patients with LUAD with low BRI *p < 0.05, ****p<0.0001.

We first conducted an overall assessment comparing the therapeutic response indicators between patients in the low-BRI and high-BRI groups (**Figure 7A**). We validated whether the BRI is associated with patient response to immunotherapy in two datasets (GSE126044 and GSE91061) that include patient outcomes of immune checkpoint blockade (ICB) therapy. Based on our prognostic model, we assigned a BRI to each patient. First, we combined the patient data from the two datasets and compared the BRI between patients with progressive disease/stable disease (PD/SD) and those with partial response/complete response (PR/CR). We found that patients in the PD/SD group had higher BRI (**Figure 7B**). Subsequently, we categorized patients in each dataset into high-BRI and low-BRI groups based on their BRI and compared the ratios of PD/SD to PR/CR between the two groups. We observed that a greater proportion of patients in the low-BRI group responded to immunotherapy (**Figure 7C**). Subsequently, our investigation focused on the IC50 metrics for standard chemotherapy and specialized therapeutic medications. Consequently, patients with LUAD and low BRI exhibited reduced IC50 values for Buparlisib (**Figure 7D**), Docetaxel (**Figure 7E**), Erlotinib (**Figure 7F**), Osimertinib (**Figure 7G**), Topotecan (**Figure 7H**), Vinorelbine (**Figure 7I**). Additionally, we constructed a nomogram based on TCGA data to predict patients’ survival (**Supplementary Figure 4**).

## 4 Discussion

Tumor immunotherapy has become the most promising cancer treatment strategy, completely transforming the landscape of lung cancer treatment. However, due to the complexity of the tumor immune microenvironment, the efficacy of immunotherapy in some lung cancer patients remains unsatisfactory (28). This study developed a BRI (biomarker-based risk index) that was identified as an independent risk factor for overall survival in patients with LUAD. The outcome of our study revealed that the BRI score is a predictive indicator in assessing immune microenvironment and the response to immunotherapy.

Mounting evidence has identified that the TILBs (tumor-infiltrating lymphocytes B cells) within the TME promotes immunosuppression and thus the associated tumor survival and progression. In ovarian cancer and melanoma, plasmablast-like TILBs express higher levels of IFNγ and chemokines (CCL3, CCL4, CCL5), which attract T cells, macrophages, and NK cells, and are indeed associated with higher T cell infiltration (29). TILBs can also promote the formation and maintenance of TLS (tertiary lymphoid structures) through the secretion of lymphotoxin α1β2 (30), which has been shown to be essential for reducing tumor growth in mouse melanoma models. Conversely, B cell-derived lymphotoxin promotes the growth of androgen-independent prostate tumors in mice. Evidence from one study demonstrated that TILBs possess tumor antigens acquired *in vivo (* 31), and CD69+ CD21+ CD27+ TILBs isolated from human lung cancer samples can stimulate autologous CD4+ TILs *in vitro (* 32, 33). However, the crosstalk of TILBs with other cells in tumors and the functions they perform require further research.

In the current study, we established a methodology to quantify the infiltration of TILBs in tumors, aiming to elucidate the interactions between TILBs and other cells within the tumor immune microenvironment, with the hope of identifying more precise personalized immunotherapies. Our study developed a BRI model using 10 different algorithms and 101 algorithm combinations within the TCGA cohort. After analyzing single-cell sequencing data, WGCNA, NMF, and univariate Cox analysis, 86 markers were ultimately selected for model construction and the final model was composed of 27 genes, which was constructed by RSF and superPC algorithms. The integration of multiple machine learning algorithms allowed for dimensionality reduction, leading to a more simplified and translatable prognostic model. The AUC values for this BRI model were 0.702 at 1 year, 0.678 at 3 years, and 0.664 at 5 years. The predictive performance of the BRI model was also validated in the GSE31210, and GSE72094 datasets. Additionally, BRI was identified as an independent risk factor for overall survival in patients with LUAD.

In subsequent analyses, patients were stratified into BRI-high and BRI-low groups based on the median BRI. Kaplan-Meier survival curve analysis indicated that the BRI-low group exhibited a higher survival rate. Through Gene Set Enrichment Analysis (GSEA), we identified that the differentially expressed genes between the two groups were primarily enriched in pathways related to B cell receptor signaling, Th1 and Th2 cell differentiation, among other tumor immune processes. Notably, B cells can modulate T cell responses under specific therapeutic conditions (34). Subsequently, we performed immune infiltration analyses using CIBERSORT, XCELL, and QUANSTEQ algorithms. The results indicated that the BRI-low group exhibited higher levels of infiltration by B cells, M2 macrophages, NK cells, CD4+ T cells, and regulatory T cells, which correlates with the previously noted better prognosis for this group. Based on these findings, we further explored the relationship between BRI and the benefits patients derive from immunotherapy. Immunotherapy based on immune checkpoints is viewed as a hopeful strategy for patients with LUAD, particularly for those in advanced phases (35). Focusing on immune checkpoint molecules like PD-1 and CTLA-4 has the potential to revitalize anti-cancer immunity (36). T cells are acknowledged as key to the effectiveness of existing cancer immunotherapies (37). Conversely, B cells exhibiting a lower BRI score demonstrate elevated TIME (tumor immune microenvironment) scores, increased CTLA4 immunophenoscores, and reduced scores in tumor immune dysfunction and exclusion, immune monitoring, and immune evasion. A higher TIDE score suggests a greater chance of immune evasion and a diminished effectiveness of ICI (immune checkpoint inhibitor) therapy (38, 39). Reduced immunophenoscore indicates an improved reaction to ICI therapies (40). Consequently, patients with LUAD and lower BRI might gain greater advantages from immunotherapy. Recognizing TILBs’ vital function in chemotherapy, our study focused on analyzing the IC50 values for both standard chemotherapy and specific therapeutic drugs. Our research found that LUAD patients with elevated BRI exhibited reduced IC50 scores for medications like Crizotinib, Savolitinib, Ulixertinib, 5-Fluorouracil, Cisplatin, Docetaxel, Gemcitabine, and Paclitaxel. This implies a higher susceptibility of these patients to chemotherapy and specialized treatments. This study is specifically designed to evaluate whether TILBs offer additional prognostic significance beyond the conventional TNM staging system, with a particular focus on patients with early-stage LUAD. The BRI may serve as a valuable tool for oncologists to identify patients who would benefit from chemotherapy drug treatment or ICB therapy, while also potentially sparing low-risk individuals from unnecessary overtreatment.

This study has several limitations that warrant consideration. Firstly, the C-index of our BRI model was not sufficiently high, indicating potential limitations in the predictive accuracy of our BRI model. This suggests the need for further validation using larger and more diverse clinical datasets to assess the model’s robustness and generalizability in real-world settings. Additionally, the roles of most BRI-associated genes in LUAD remain unclear, highlighting the need for further *in vivo* and *in vitro* studies to elucidate their mechanisms of action and functional significance, particularly in the context of potential challenges for clinical application, such as the model’s complexity and the implications of the low C-index. Future research should address these gaps to enhance the clinical utility of the BRI model.

## Data Availability

The raw data supporting the conclusions of this article will be made available by the authors, without undue reservation.

## Supplementary material

Supplementary Figure 1 The accuracy of cell annotation was assessed using marker genes of various cell types.

Supplementary Figure 2 Identification of key modules associated with clinical outcomes in the TCGA-LUAD dataset was conducted using WGCNA. **(A)** The analysis involved assessing the scale-free fit index and mean connectivity across various soft-thresholding power values, with a target fit index of 0.85. **(B)** A heatmap depicted gene interactions between modules, with different colors representing various modules on the axes and varying shades of yellow indicating the strength of interactions. **(C)** The WGCNA algorithm identified five co-expression modules, each shown as a branch in the dendrogram, grouping genes with similar expression patterns. **(D)** Another heatmap illustrated the correlation between these modules and clinical outcomes such as survival time and status, highlighting that genes in the turquoise and blue modules had the most significant positive and negative correlations with clinical outcomes and were selected for further investigation.

Supplementary Figure 3 The differentially expressed genes between BRI-high and BRI-low were primarily enriched in pathways related to B cell receptor signaling,Th1 and Th2 cell differentiation, Intestinal immune network for IgA production, Primary immunodeficiency, Th17 cell differentiation in LUAD, GSE31210 and GSE72094 cohorts.

Supplementary Figure 4 In the TCGA-LUAD cohort, a nomogram was constructed incorporating patient age, gender, tumor stage, and BRI to predict the 1-year, 3-year, and 5-year survival rates.
