# Supplementary figures and images for: A tumor-infiltrating B lymphocytes -related index based on machine-learning predicts prognosis and immunotherapy response in lung adenocarcinoma
Source: Front Immunol. 2025 Mar 24;16:1524120. doi: 10.3389/fimmu.2025.1524120 (PMC11973313; doi:10.3389/fimmu.2025.1524120)

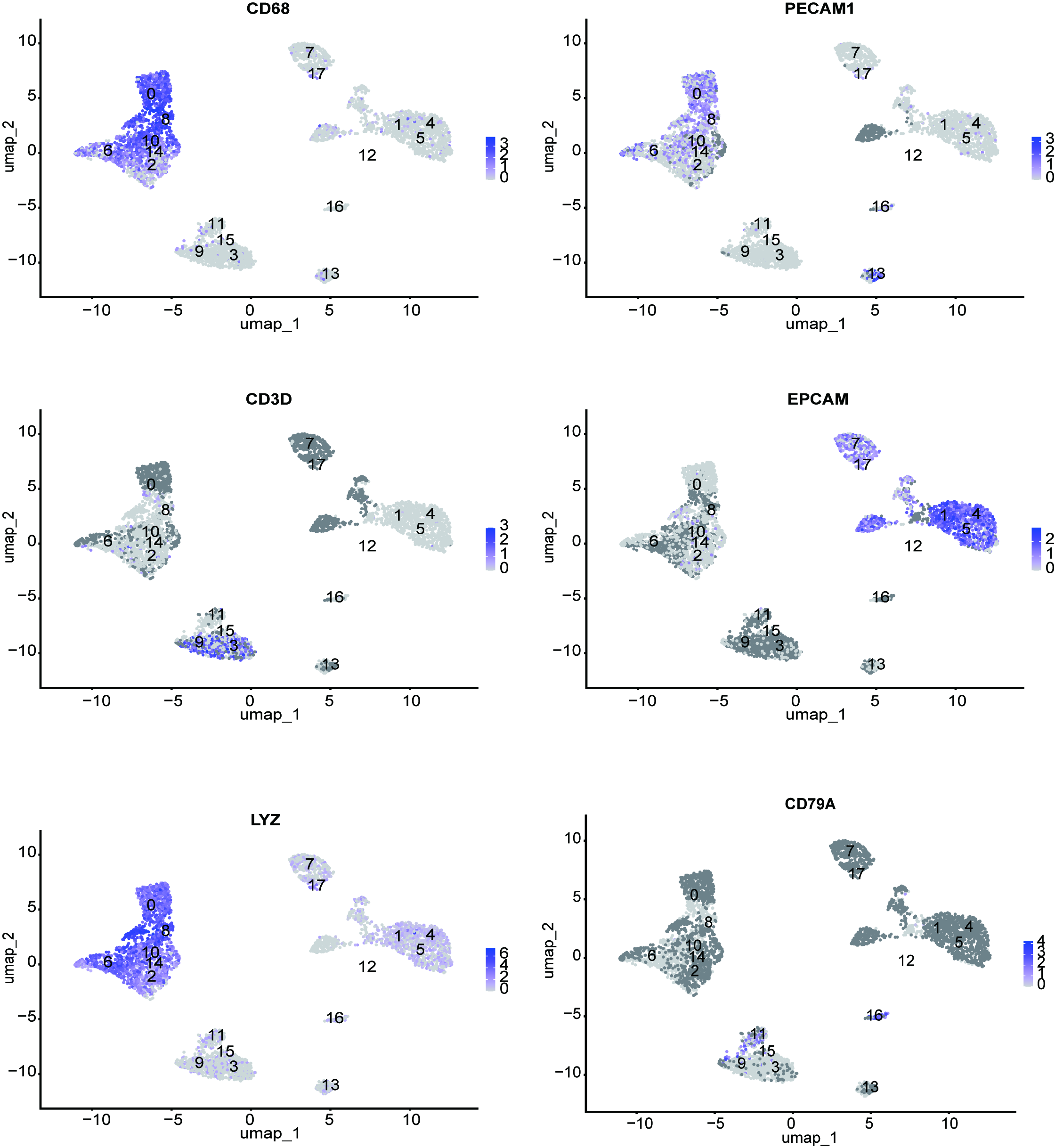

Supplement: Supplementary Figure 1 — The accuracy of cell annotation was assessed using marker genes of various cell types. [file Image1.tif]

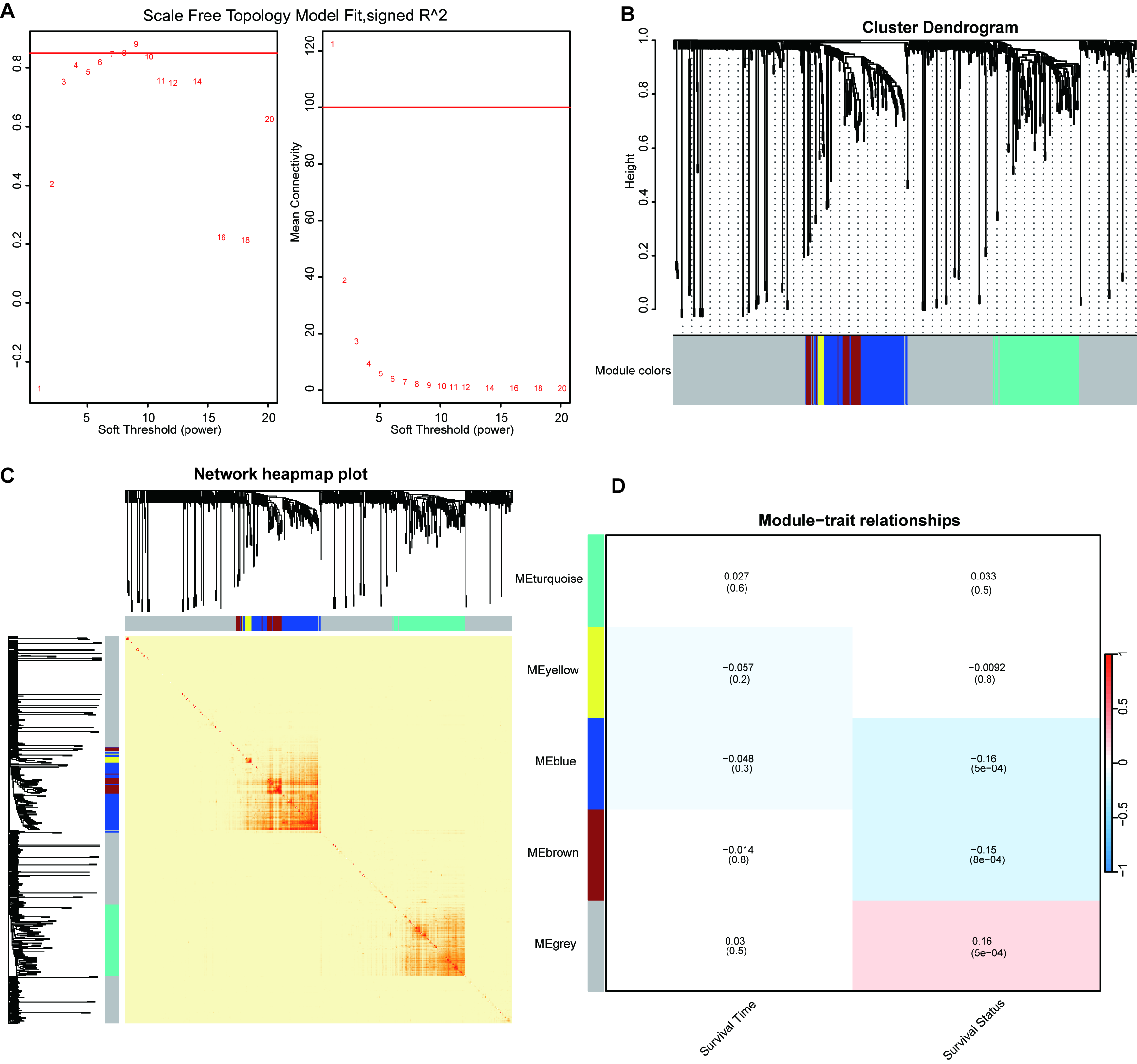

Supplement: Supplementary Figure 2 — Identification of key modules associated with clinical outcomes in the TCGA-LUAD dataset was conducted using WGCNA. (A) The analysis involved assessing the scale-free fit index and mean connectivity across various soft-thresholding power values, with a target fit index of 0.85. (B) A heatmap depicted gene interactions between modules, with different colors representing various modules on the axes and varying shades of yellow indicating the strength of interactions. (C) The WGCNA algorithm identified five co-expression modules, each shown as a branch in the dendrogram, grouping genes with similar expression patterns. (D) Another heatmap illustrated the correlation between these modules and clinical outcomes such as survival time and status, highlighting that genes in the turquoise and blue modules had the most significant positive and negative correlations with clinical outcomes and were selected for further investigation. [file Image2.tif]

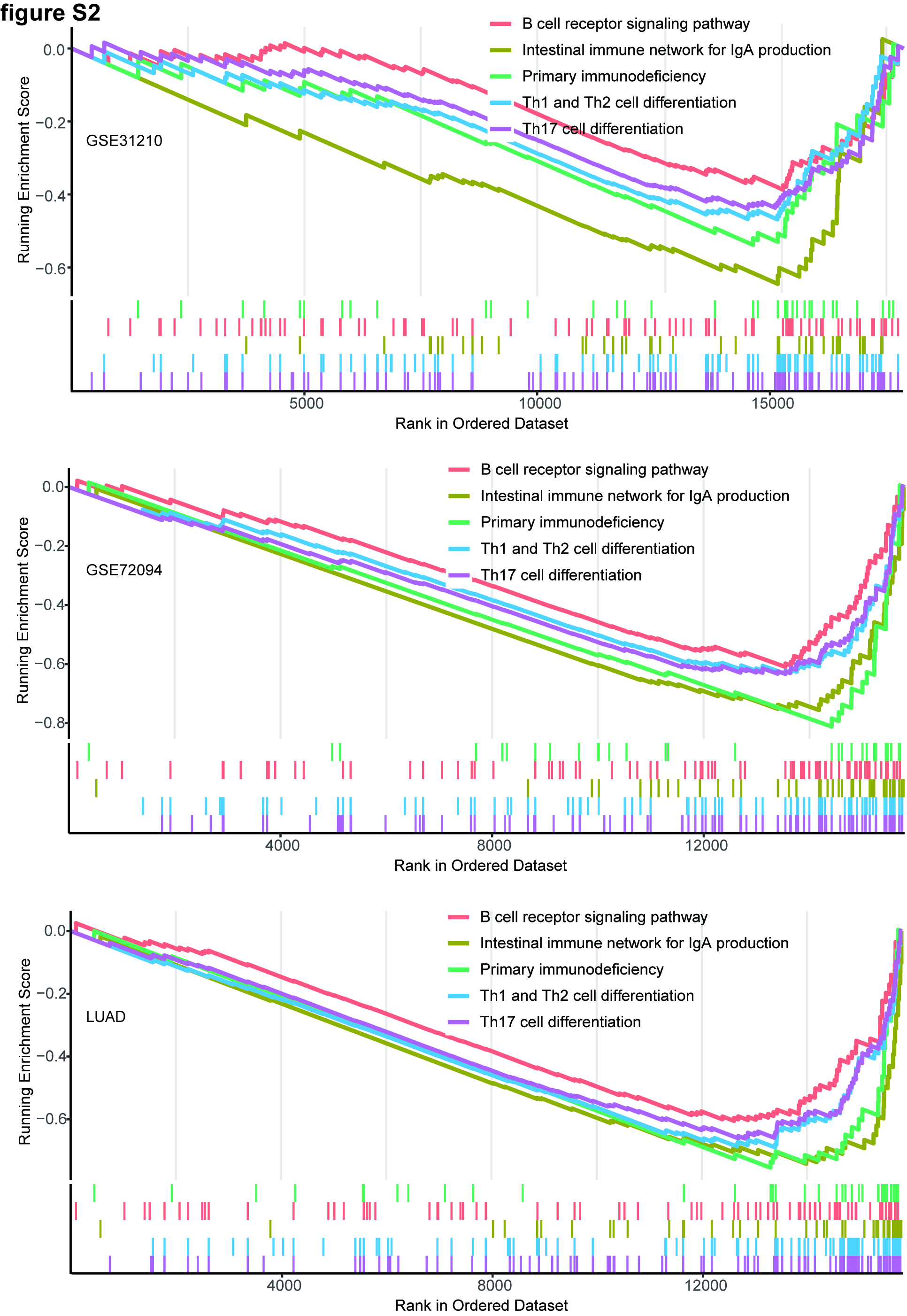

Supplement: Supplementary Figure 3 — The differentially expressed genes between BRI-high and BRI-low were primarily enriched in pathways related to B cell receptor signaling,Th1 and Th2 cell differentiation, Intestinal immune network for IgA production, Primary immunodeficiency, Th17 cell differentiation in LUAD, GSE31210 and GSE72094 cohorts. [file Image3.tif]

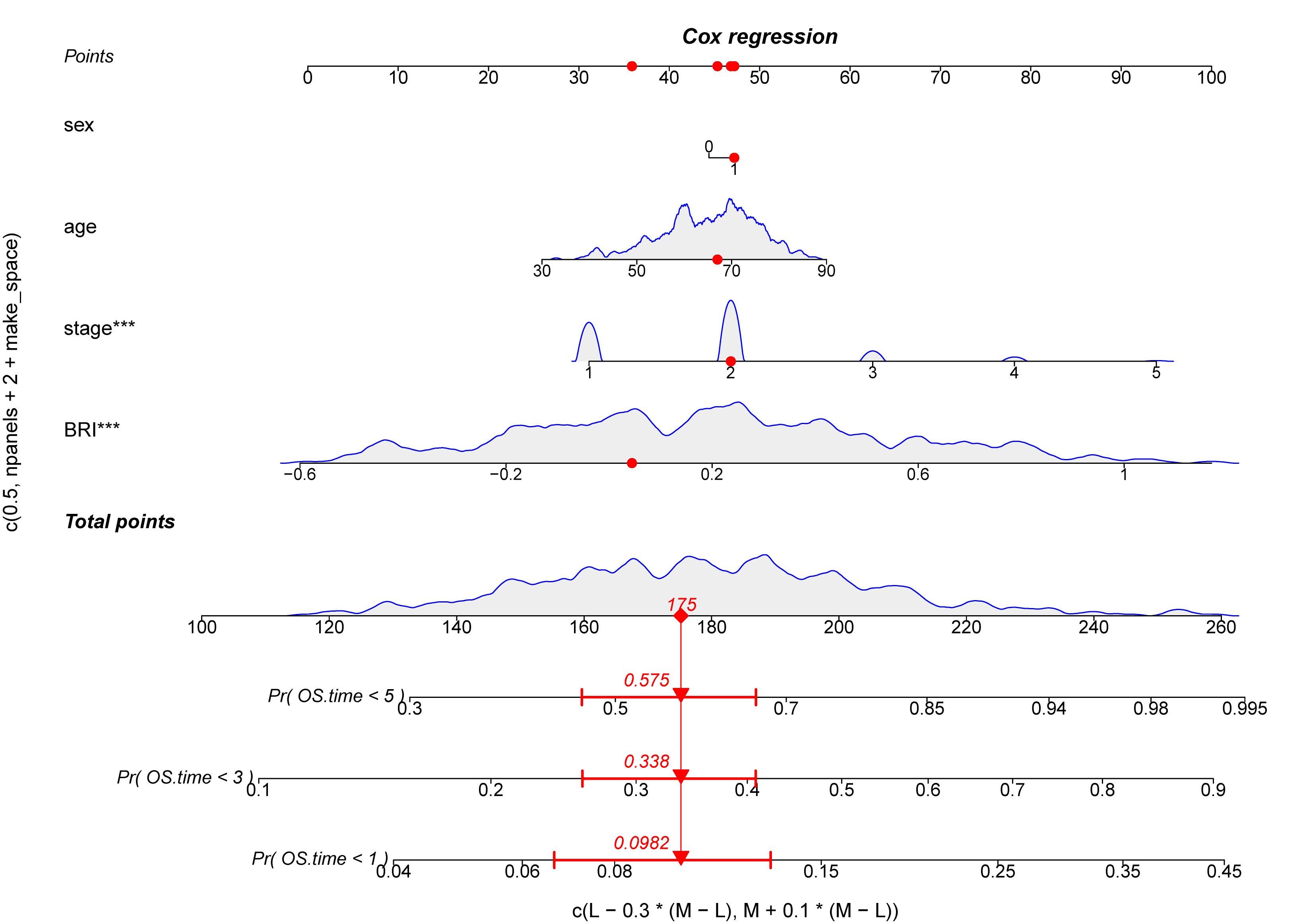

Supplement: Supplementary Figure 4 — In the TCGA-LUAD cohort, a nomogram was constructed incorporating patient age, gender, tumor stage, and BRI to predict the 1-year, 3-year, and 5-year survival rates. [file Image4.tif]
